# Antitumor activity of TY-011 against gastric cancer by inhibiting Aurora A, Aurora B and VEGFR2 kinases

**DOI:** 10.1186/s13046-016-0464-2

**Published:** 2016-11-25

**Authors:** Wang Liu, Yu Lu, Xiaoping Chai, Xiao Liu, Tong Zhu, Xihan Wu, Yanfen Fang, Xuan Liu, Xiongwen Zhang

**Affiliations:** 1 Shanghai Engineering Research Center of Molecular Therapeutics and New Drug Development, School of Chemistry and Molecular Engineering, East China Normal University, Shanghai, China; 2 Nanjing Tianyi Bioscience Co. Ltd, Nanjing, China; 3 Department of Cardiology, Shanghai Renji Hospital, School of Medicine, Shanghai Jiaotong University, Shanghai, China; 4 School of Physics and Materials Science, East China Normal University, Shanghai, China

**Keywords:** Gastric cancer, Cell cycle, Angiogenesis, Polyploidy, Apoptosis

## Abstract

**Background:**

Overexpression of Aurora A and B has been reported in a wide range of tumor types, including gastric cancer. Anti-angiogenesis has been considered as an important therapeutic modality in advanced gastric cancer. Here we identified a novel compound TY-011 with promising antitumor activity by targeting mitotic kinases (Aurora A and B) and angiogenic receptor tyrosine kinase (VEGFR2).

**Methods:**

HTRF® KinEASE™ assay was used to detect the effect of TY-011 against Aurora A, Aurora B and VEGFR2 activities. Docking simulation study was performed to predict the binding mode of TY-011 with Aurora A and B kinases. CCK-8 assay was used to test cell growth. Cell cycle and cell apoptosis was analyzed by flow cytometry. Gastric cancer cell xenograft mouse models were used for in vivo study. TUNEL kit was used to determine the apoptosis of tumor tissues. Immunohistochemistry analysis and HUVEC tube formation assay were performed to determine the anti-angiogenesis ability. Immunofluorescence and western blot were used to test protein expression.

**Results:**

TY-011 was identified as a potential Aurora A and B inhibitor by HTRF® KinEASE™ assay. It effectively inhibited cellular Aurora A and B activities in a concentration-dependent manner. TY-011 occupied the ATP-binding site of both Aurora A and B kinases. TY-011 demonstrated prominent inhibitory effects on proliferation of gastric cancer cells. TY-011 treatment induced an obvious accumulation of cells at G2/M phase and a modest increase of cells with >4 N DNA content, which then underwent apoptosis. Meaningfully, orally administration of TY-011 demonstrated superior efficacy against the tumor growth in gastric cancer cell xenograft, with ~90% inhibition rate and 100% tumor regression at 9 mg/kg dose, and TY-011 did not affect the body weight of mice. Interestingly, we observed that TY-011 also antagonized tumor angiogenesis by targeting VEGFR2 kinase.

**Conclusions:**

These results indicate that TY-011 is a well-tolerated, orally active compound that targets mitosis and angiogenesis in tumor growth, and provides strong preclinical support for use as a therapeutic for human gastric cancers.

**Electronic supplementary material:**

The online version of this article (doi:10.1186/s13046-016-0464-2) contains supplementary material, which is available to authorized users.

## Background

Gastric cancer (GC) as a highly heterogeneous disease is the fifth most common malignancies in the world and accounts for more than half of cases annually in East Asia [1, 2]. Unfortunately, most gastric cancers are diagnosed in locally advanced or metastatic stage, which is related to a poor prognosis [3]. Chemotherapy remains the main treatment for advanced GC and seems to have reached an efficacy plateau [4]. Multiple oncogenic signaling pathways have been shown to regulate cancer cell survival and drug resistance against chemotherapies, however, the survival rate for patients with GC has not shown any significant improvement yet [5, 6]. There is an urgent need for finding new treatments and strategies to improve outcomes.

During mitosis, the Aurora kinases are a family of serine/threonine kinases that play crucial roles for cell cycle control. The Aurora family has three members: Aurora A, B and C, which are very similar within the carboxy-terminal catalytic domain, but shows little similarity in N-terminus [7, 8]. Aurora A and B have attracted plenty of attention as they are closely related with occurrence of cancer. Aurora A plays an important role in centromere duplication and maturation throughout the late G2 to mitotic phases, therefore Aurora A inhibition delays initiation of mitosis, which in turn causes mitotic cells accumulation [9]. In contrast, Aurora B ensures the integrity of chromosome segregation at the spindle assembly checkpoint (SAC) during metaphase, and executes cytokinesis in anaphase and telophase. Aurora B inhibition causes inactivation of the SAC and accelerates mitotic slippage, creating polyploidy cells [10, 11]. In addition, Aurora A has also been proposed to play a cooperative role in cytokinesis recently [12, 13]. Therefore, both Aurora A and B contribute to accurate chromosome segregation during mitotic exit.

Aurora A and B are overexpressed in a wide range of tumor types, including GC [14, 15]. Overexpression of Aurora A has been shown to promote cell survival through regulation of HDM2 in gastric cancer cell lines [16], promote inflammation and tumorigenesis by activating NF-κB through directly phosphorylation of IκBα in mice and human gastric cancer [17]. Overexpression of Aurora B might contribute to DNA aneuploidy by promoting chromosomal instability in human gastric cancers [18]. Moreover, previous study has shown that Aurora A deletion inhibits the proliferation, viability, migration and invasion of gastric cancer cells [19]. Aurora A inhibitor MLN8237 has been shown to induce apoptosis and autophagy of human gastric cancer cells in vitro and reduce tumor growth in xenograft tumor model using gastric cancer cells [16, 17, 20]. All these evidences indicated that Aurora kinases are potential therapeutic targets in gastric cancer.

Tumor angiogenesis has long been considered as an attractive therapeutic target for cancer treatment [21]. VEGF is one of the most important and potent angiogenic factors, and is closely linked with growth and metastatic spreading of several types of tumors [22]. High levels of VEGF concentrations have been reported to relate with vascular dissemination and poor outcomes in patients affected by GC [23, 24]. As the major receptor of VEGF, VEGFR-2 has been considered as an important therapeutic modality in advanced gastric cancer. Apatinib, a small-molecule tyrosine kinase inhibitor that strongly inhibits VEGFR2, has significantly improved the median overall survival of GC patients who progressed to at least two lines of chemotherapy compared with placebo in a phase III clinical trial [25]. Ramucirumab, a monoclonal antibody binding to VEGFR2, is currently recommended in GC patients progressing after first-line treatment [6]. Therefore, VEGFR pathways have attracted great interest in GC therapy.

**Fig. 1:**
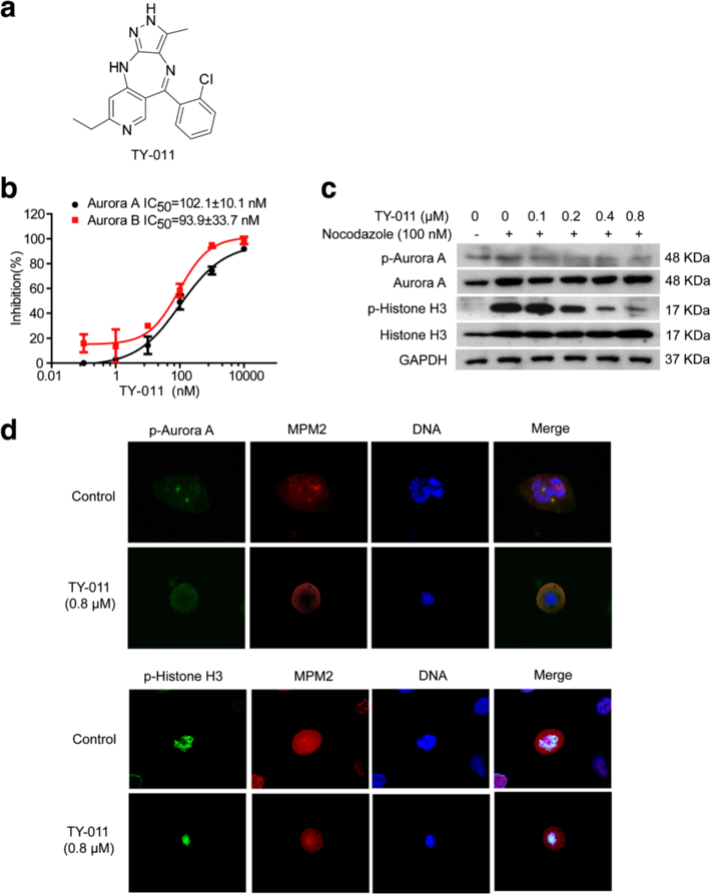
TY-011 inhibits Aurora A and Aurora B kinases activities. **a** Chemical structure of TY-011. **b** The HTRF® KinEASE™-STK kit was used to measure inhibition of Aurora A and Aurora B activities by TY-011. The inhibition rates were determined in reference to the control. Data presented are the mean ± SD of three independent experiments. **c** Effects of TY-011 on phosphorylation of Aurora A and Histone H3 were analyzed by western blot. MGC-803 cells synchronized with nocodazole (100 nM) for 16 h were treated with various concentration of TY-011 for 24 h, then were lysed and subjected for western blot. The results are representative of three independent experiments. **d** Effects of TY-011 on phosphorylation of Aurora A and Histone H3 were analyzed by immunofluorescence. MGC-803 cells were treated with TY-011 (0.8 μM) for 24 h, then fixed and stained for p-Aurora A (T288) (*green*) or p-Histone H3 (H10) (*green*), MPM2 (*red*) and DNA (*blue*). MPM2 was used to locate the mitotic cells. The images are representative of two independent experiments

We have synthesized a series of compounds and evaluated their activities against Aurora A and Aurora B kinases by HTRF® KinEASE™-STK assay. A novel compound TY-011 (Fig. 1a) was identified to have high activity against Aurora A and B kinases, with IC_50_ of about hundreds nanomole. Cell biological experiments further confirmed that TY-011 potently inhibited cellular activities of these two kinases. The molecular docking analysis demonstrated that TY-011 occupied the ATP-binding site of both Aurora A and B kinases. Importantly, TY-011 exhibited potential activity against gastric cancer in vitro and in vivo. Out of expectation, we observed that TY-011 also antagonized tumor angiogenesis by targeting VEGFR2 kinase. Together, these evidences indicate that TY-011 works as a dual kinases inhibitor that targets mitosis progression and angiogenesis, and provide strong confidence for further development for GC therapy.

## Methods

### Reagents

TY-011 (9-(2-chloro-phenyl)-6-ephyl-1-methyl-2,4-dihydro-2,3,4,7,10-pentaaza-benzo[f]azulene), with purity >98%, was synthesized by Nanjing Tianyi Bioscience Co. Ltd, (Nanjing, Jiangsu, China). The compound was dissolved in DMSO as 50 mM stock solution for enzymatic assays and cell based assays. For animal studies, TY-011 was dissolved in 5% Klucel EF + 5% ethanol +90% normal saline, the stock solution was freshly prepared every 3 days. Irinotecan was purchased from Biocompounds Pharmaceutical Inc. (Shanghai, China) and was dissolved in sterile water.

### Cell culture

The human GC cell lines (AGS, MGC-803, MKN-45, SGC-7901 and SNU-16) were purchased from Cell Bank of China Science Academy (Shanghai, China) and maintained in RPMI-1640 or Ham’s F-12 K medium supplemented with 10% FBS (PAN Biotech, Bavaria, Germany). Human umbilical vein endothelial cell (HUVEC) obtained from Allcells (Emeryville, CA, USA) were kept in HUVEC basic medium supplemented with 10% FBS (Allcells, Emeryville, CA, USA) and endothelial cell growth supplements. Penicillin (100 U/ml) and streptomycin (100 U/ml) were added in the medium. All these cells were maintained in an atmosphere of 5% CO2 and 95% air at 37 °C.

### Molecular modeling

X-ray protein structure of Aurora A (PDB ID: 4ZTQ) and Aurora B (PDB ID: 4AF3) were obtained in Protein Data Bank. All water molecules in the crystal structure were removed, and then the structure was used for molecular docking using Sybyl *X*2. For docked structure, energy minimization and molecular dynamic (MD) simulation were performed using the Sander module in Amber14. A two-step, extensive energy minimization process based on the steepest descent method followed by the conjugate gradient algorithm were carried out to relieve bad contacts and to direct each system toward energetically favorable conformations. After minimization, each system was gently heated from 0 to 300 K in 500 ps at constant volume and equilibrated at 300 K for another 500 ps. Finally, a 30 ns MD simulation without any restrictions was performed at constant pressure, and the coordinates of atoms were saved every 2 ps. During the MD simulation, all bonds involving hydrogen atoms were constrained using the SHAKE algorithm, and a time step of 2 fs was adopted. The temperature was controlled using the Langevin thermostat with a collision frequency of 2.0 ps − 1. The particle mesh Ewald (PME) method was applied to treat the long-range electrostatic interactions. The cutoff distances for the long-range electrostatic and van der Waals interactions were set to 12.0 Å. Solvated interaction energy (SIE) method was used to calculate the binding free energies between compounds and Aurora A/B. For the current work, 200 snapshots extracted from the last 10 ns of MD trajectory at an interval of 50 ps were used for the binding free energy analyses.

### Kinase assays

The inhibition of TY-011 on activity of Aurora A, Aurora B and VEGFR2 was characterized by HTRF® KinEASE™-STK/TK kit according to the manufacturer’s instructions (Cisbio Assays, Codolet, France). Aurora A and Aurora B were gifts from Professor Jia Li, Shanghai Institute of Materia Medica/Chinese Academy of Sciences. VEGFR2 was purchased from Carna Biosciences (Kobe, Japan).

### Cell proliferation assay

During the logarithmic growth phase, each cell line was seeded in a 96-well tissue culture plate (Thermo Fisher Scientific, Waltham, MA, USA) at a predetermined density in complete medium, attached overnight and treated with compound for the indicated time. The medium was discarded and replaced with 10% CCK-8 (Dojindo, Kumamoto, Japan) in complete medium, then incubated the plates for another 2 h. The OD450 was measured with SpectraMax M5 (Molecular Devices, CA, USA). A background absorbance of OD_blank_ was subtracted from all wells. The survival rate was determined with following formula: Survival rate (%) = OD_compound_/OD_DMSO_ × 100%. The IC_50_ values were calculated by concentration − response curve fitting using the four parameter method.

In VEGF-induced HUVEC proliferation assay, HUVECs cells were seeded in a 96-well tissue culture plate at a predetermined density in HUVEC basic medium with 0.5% FBS. After the cells attached, cells were treated with different concentration of TY-011 in the presence of 50 ng/ml VEGF165 (R&D systems, Minneapolis, MN, USA) or 10% FBS. The CCK-8 assay as above was used for determination of cell viability.

### Cell cycle analysis

Cell cycle distribution was determined by flow cytometry measurement of DNA content after cells were incubated with RNase A (50 μg/ml) and propidium iodide (50 μg/ml). The cellular DNA content was measured on a flow cytometer (Guava EasyCyte 6HT-2 L, Merck Millipore, Billerica, MA, USA). The percentage of each population was analyzed using the InCyte software. At least 20 000 cells were analyzed for each data point.

### Apoptosis analysis

Apoptosis was detected by FITC Annexin V Apoptosis Detection Kit I according to the manufacturer’s instructions (Dojindo, Kumamoto, Japan) to determine the phosphatidyl serine exposure. Apoptotic cells were quantified by a flow cytometer (Guava EasyCyte 6HT-2 L, Merck Millipore, Billerica, MA, USA) using the InCyte software. At least 10 000 cells were analyzed for each data point.

### Western blot

The proteins of whole cell lysates were prepared in RIPA buffer containing protease and phosphatase inhibitors (Thermo Fisher Scientific, Waltham, MA, USA), and quantified by the bicinchoninic acid (BCA) method. Immunoblotting analysis of proteins in cell lysates was performed as previously described [26]. Primary antibodies used were as follows: anti-Aurora A (#12100), anti-p-Aurora A (Thr288) (#3079), anti-Histone H3 (#4499), and anti-p-Histone H3 (Ser10) (#9701), and anti-cleaved caspase 3 (#9661) were purchased from Cell Signaling Technology (Danvers, MA, USA); anti-PARP-1 (#sc-8007) and anti-GAPDH (#sc-25778) were purchased from Santa Cruz (CA, USA).

### Immunofluorescence

Cells were cultured on glass slides coated with 0.05 mg/ml PDL (Sigma-aldrich, St. Louis, MO, USA) in the presence of TY-011 (0.8 μM) or equivalent amount of DMSO for 24 h. The cells were fixed in 4% paraformaldehyde at room temperature, then permeabilized with 0.15% Triton-X100 and blocked in 3% BSA in PBS. The primary antibodies were incubated with slides at 1:100 dilution in PBS containing 3% BSA in a wet chamber at 4 °C overnight. The secondary antibodies Alexa Fluor H 488 goat anti-rabbit IgG (H + L) or Alexa Fluor H 594 goat anti-mouse IgG (H + L) (Invitrogen, Carlsbad, CA, USA) were applied respectively for 2 h after the slides were washed with PBS thoroughly at room temperature. After the unbound antibodies were removed, the slides were dried and mounted with DAPI-fluoromount-G (Beyotime, Shanghai, China). The images were captured with FCFM (fluorescence confocal microscope) (Olympus FV1000 IX81, Tokyo, Japan) under appropriate excitation and emission filters or phase-contrast microscope (Olympus IX73, Tokyo, Japan). Primary antibodies used were as follows: anti-p-Aurora A (Thr288) (#3097) and anti-p-Histone H3 (Ser10) (#9701) were purchased from Cell Signaling Technology (Danvers, MA, USA); anti-MPM2 (anti-phospho-Ser/Thr-Pro) (#05-368) was purchased from Merck Millipore (Billerica, MA, USA); and anti-α-tubulin (sc-8035) was purchased from Santa Cruz (CA, USA).

### HUVEC tube formation assay

HUVECs were seeded onto pre-cooled 96-well tissue culture plate coated with Matrigel™ (BD Biosciences, FranklinLakes, NJ). After the cells attached, the cells were treated by various concentration of TY-011 or equivalent amount of DMSO. The plate was incubated at 37 °C, 5% CO_2_ humidified atmosphere for 6 h, then cell morphology was captured with a microscopy (Olympus IX73, Tokyo, Japan).

### Antitumor activity in vivo

Female BALB/c nu/nu mice (5–6 weeks, 16–18 g) were obtained from Sino-British SIPPR/BK Lab. Animal Ltd, Shanghai, China, with the certification number of 2008001638201. The animals were housed in specific pathogen-free (SPF) conditions at Key Laboratory of Brain Functional Genomics, Ministry of Education, and were acclimatized for a week prior to use. Human MGC-803 xenografts were established by subcutaneously inoculating 5 × 10^6^ cells into nude mice. When the tumors reached a mean group size of 100 ~ 150 mm^3^, the mice were randomized into control and treatment groups to receive treatment accordingly. TY-011 was orally administered at 3, 6 and 9 mg/kg once a day for 13 days. Irinotecan was intraperitoneally administered at 50 mg/kg twice a week and served as positive control of this experiment. The tumor growth was recorded with the measurement of length (L) and width (W) by caliper every other day, and calculated as tumor volume (V) = L × W^2^/2. Meanwhile, the body weights of mice were recorded. The tumor volume at day *n* was expressed as relative tumor volume (RTV) according to the following formula: RTV% = (TV_*n*_/TV_0_) ×100%, where TV_*n*_ is the tumor volume at day *n* and TV_0_ is the tumor volume at day 0. The therapeutic effect of treatment was calculated with formula T/C (%) = mean RTV of the treated group/mean RTV of the control group × 100%. The tumor growth inhibition was calculated as TGI% = (1-(mean tumor volume of the treated group on the first day-mean tumor volume of the treated group on the end day)/ (mean tumor volume of the control group on the first day-mean tumor volume of the control group on the end day)) ×100%. The inhibition rate = (1-mean tumor weight of the treated group/ mean tumor weight of the control group) ×100%. The tumor regression of individual mouse was defined as the tumor volume at the end was less than the tumor volume when treatment was initiated.

### Immunohistochemistry

Tumor tissues from mice were embedded in a paraffin block and subjected to immunohistochemistry. Tumor tissues were deparaffinized and hydrated, then permeabilized with 0.5% Triton X-100/1 PBS for 10 min, hybridized with the rabbit anti-human CD31 polyclonal antibody (sc-8306; 1:200; Santa Cruz) and an HRP-conjugated goat anti-rabbit antibody was used as the secondary antibody. After developing with substrate-chromogenic solution (DAB, DAKO, Glostrup, Denmark), the sections were counterstained with hematoxylin. Images were captured with a microscopy (Olympus IX73, Tokyo, Japan).

### TUNEL histology

Apoptotic tumor tissue slides were detected by terminal deoxynucleotidyl transferase- deoxyuridine triphosphate nick end labeling (TUNEL) staining (DeadEnd™ Fluorometric TUNEL System, Promega, Madison, WI, USA) according to the manufacturer protocol. The slides were observed under the fluorescence microscopy (Olympus IX73, Tokyo, Japan).

### Statistical analysis

The statistical significance of differences between groups was evaluated by the unpaired Student’s *t* test and indicated with ***P < 0.001, **P < 0.01, *P < 0.05. All statistical tests were two sided.

## Results

### TY-011 inhibits activities of Aurora A and Aurora B kinases

A series of compounds was synthesized and analyzed for their activities against Aurora A and Aurora B kinases. Eight of them were found to have activities against either one of the kinases or both kinases with IC_50_ from hundreds nanomole to micromole (Additional file 1: Table S1). Of note, compound TY-011 was identified to have the best activity against Aurora A and B kinases, with IC_50_ values of 102.1 ± 10.1 nM and 93. 9 ± 33.7 nM, respectively (Fig. 1b). We then determined the proliferation inhibitory effects of these eight compounds against gastric cancer MGC-803 cells. Three of them (including TY-011) exhibited potential activities against proliferation of MGC-803 cells with IC_50_ values of 0.2-0.4 μM. Considering the potential inhibitory activity against Aurora A and B kinases as well as proliferation of MGC-803 cells, TY-011 was therefore chosen for our further studies.

We then determined whether TY-011 could inhibit intracellular activities of Aurora A and Aurora B. As Aurora kinases are expressed and active at the highest level during G2/M phase of the cell cycle [27], MGC-803 cells were arrested in G2/M phase by nocodazole treatment, which was accompanied with the increased phosphorylation of Aurora A (T288) and its total protein level. With the treatment of TY-011, the p-Aurora A (T288) decreased in a concentration-dependent manner (Fig. 1c). The phosphorylation of Histone H3 (S10), which is known to be a substrate of Aurora B, was also strongly inhibited by TY-011 in a concentration-dependent manner (Fig. 1c). Meanwhile the total protein levels of Aurora A and Histone H3 were not altered by TY-011.

Moreover, Aurora kinase inhibition by TY-011 was visualized by immunofluorescence. Phospho-Ser/Thr-Pro Mitotic protein monoclonal #2 (MPM2) was used as a mitotic marker (red) for labeling mitotic cells. Baseline evaluation showed detectable levels of p-Aurora A (T288) and p-Histone H3 (S10) in cells. In detail, the phosphorylated Aurora A (T288) exists in the centrosome of mitotic cells (green) and the phosphorylation of Histone H3 (S10) was primary localized in the centromeres of cells during mitotic. After cells were treated with 0.8 μM TY-011 for 24 h, the Aurora A green foci in mitotic cells became weaker or even disappeared (Fig. 1d, upper panel), while the p-Histone H3 (S10) decreased sharply (Fig. 1d, lower panel). Therefore, these results indicated that TY-011 could effectively inhibit the in vitro and cellular kinase activities of Aurora A and Aurora B.

### The binding mode of TY-011 with Aurora A and Aurora B

**Fig. 2:**
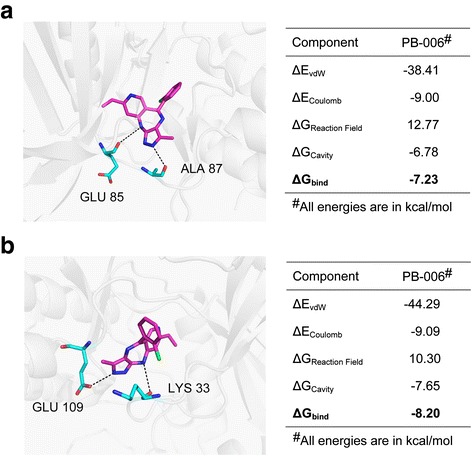
Computational predicted binding mode of TY-011 with Aurora A and Aurora B. **a** Proposed binding mode of TY-011 with Aurora A in the last snapshot of 30 ns molecular dynamic simulations. **b** Proposed binding mode of TY-011 with Aurora B in the last snapshot of 30 ns molecular dynamic simulations. Bind free energies of TY-011 to Aurora A or Aurora B were calculated by the SIE method. ΔG_bind_ is the calculated binding free energy; ΔE_vdW_ and ΔE_Coulomb_ are contributions of van der Waals and Coulomb interactions to ΔG_bind_, ΔG_Reaction Field_ and ΔG_Cavity_ describe differences in the reaction field energy and molecular surface area upon inhibitor binding

We then conducted the molecular docking analysis using Sybyl *X*2 to interpret the binding mode of TY-011 with Aurora A and B, respectively. The proposed binding modes showed that TY-011 occupied the ATP-binding site of Aurora A and Aurora B, respectively (Fig. 2 and Additional file 1: Figure S1). TY-011 formed two conserved hydrogen bond with Aurora A, one was between the nitrogen atom of pyrazole and ALA87, the other was between the amine group of the seven-member heterocycle and GLU85 (Fig. 2a). Similarly, TY-011 formed two conserved hydrogen bond with Aurora B, one was between nitrogen atom of pyrazole and GLU109, the other was between the amine group of the seven-member heterocycle and LYS33 (Fig. 2b). The calculated binding free energy was −7.23 kcal/mol and −8.20 kcal/mol between TY-011 and Aurora A as well as between TY-011 and Aurora B, respectively.

### TY-011 inhibits proliferation of cancer cells and induces cell-cycle arrest and polyploidy

**Fig. 3:**
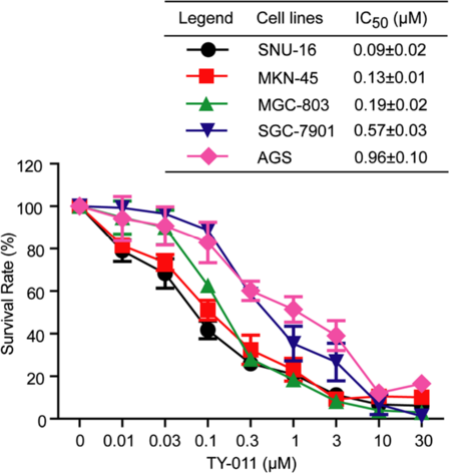
TY-011 suppresses the proliferation of gastric cancer cells. Growth inhibition assays of TY-011 in vitro were performed using the CCK-8 assay. The inhibition rates were determined in reference to the control. The concentration–survival rate curves of TY-011 on gastric cancer cells were drawn, and the IC_50_ was calculated. Data presented are the mean ± SD of three independent experiments

Given the important roles of Aurora kinases in cellular proliferation, we then evaluated the anti-proliferation activity of TY-011 against 5 gastric cancer cell lines. Concentration–inhibition curves were drawn and IC_50_s were calculated as shown in Fig. 3. TY-011 effectively suppressed cell growth in a concentration-dependent manner, with IC_50_ values ranging from 0.09 to 0.96 μM.

**Fig. 4:**
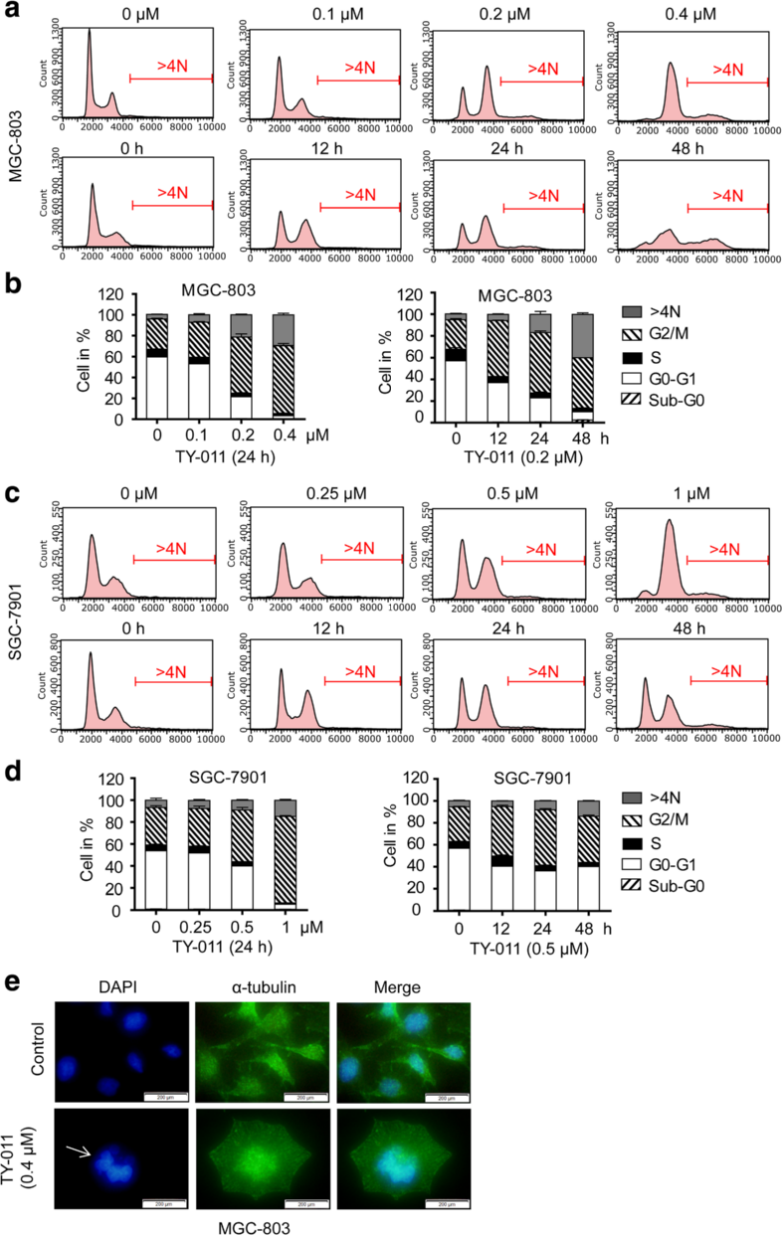
TY-011 causes accumulation of cells with ≥4 N DNA content. **a** MGC-803 cells were treated with indicated concentration of TY-011 for 24 h (*upper panel*) or with 0.2 μM TY-011 for various times (*lower panel*). Cell cycle distribution was assessed by flow cytometry after cells were stained with propidium iodide. Representative data were shown. **b** Cell cycle proportion in (**a**) was quantitated. Data presented are the mean ± SD of three independent experiments. **c** SGC-7901 cells were treated with indicated concentration of TY-011 for 24 h (*upper panel*) or with 0.5 μM TY-011 for various times (*lower panel*). Cell cycle distribution was assessed by flow cytometry after cells were stained with propidium iodide. Representative data were shown. **d** Cell cycle proportion in (**c**) was quantitated. Data presented are the mean ± SD of three independent experiments. **e** MGC-803 cells were treated with 0.4 μM TY-011 for 24 h, and stained with anti-α-tubulin and DAPI for microtubules and chromosome, respectively. Scale bar, 20 μm

Inhibition of Aurora A induced cell cycle G2/M phase arrest [12], while suppression on Aurora kinase B is reported to cause polyploidy in cells without cytokinesis [13]. The effects of TY-011 on cell cycle distribution was then determined in MGC-803 and SGC-7901 cells. In both cells, TY-011 treatment induced an obvious accumulation of cells in G2/M phase and with >4 N DNA content at 24 h, in a concentration-dependent manner (Fig. 4a-d). In detail, the proportion of G2/M phase cells increased from 28.8% with no treatment to 65.0% with 0.4 μM TY-011 treatment, and the proportion of cells with >4 N DNA content increased from 4.3 to 29.5% in MGC-803 cells (Fig. 4a and b). Similarly, the proportion of G2/M phase cells increased from 34.0% with no treatment to 78.6% with 1 μM TY-011 treatment, and the proportion of cells with >4 N DNA content increased from 6.7 to 14.7% in SGC-7901 cells (Fig. 4c and d).

The effects of TY-011 on cell cycle distribution at different time intervals were further evaluated. In both MGC-803 and SGC-7901 cells, a significant accumulation of G2/M phase cells was detected at 12 h after TY-011 treatment (from 26.4 to 51.4% in MGC-803 cells and from 31.6 to 45.1% in SGC-7901 cells), which increased a little at the following 12 h, and then decreased slightly at 48 h. Meanwhile, the population of cells with >4 N DNA content started to accumulate at 24 h, albeit slightly, and increased to 39.8 and 13.9% at 48 h in MGC-803 and SGC-7901 cells, respectively (Fig. 4a–d).

Moreover, TY-011-induced polyploidy was further confirmed by microscopic inspection. Compared with control cells, chromatins of cells treated with TY-011 were not segregated properly, therefore leading to formation of polyploidy (Fig. 4e). Together, these results showed that TY-011 induced G2/M phase arrest and polyploidy by inhibiting Aurora A and Aurora B kinases, thus inhibiting the proliferation of cancer cells.

### TY-011 triggers apoptosis of cancer cells

**Fig. 5:**
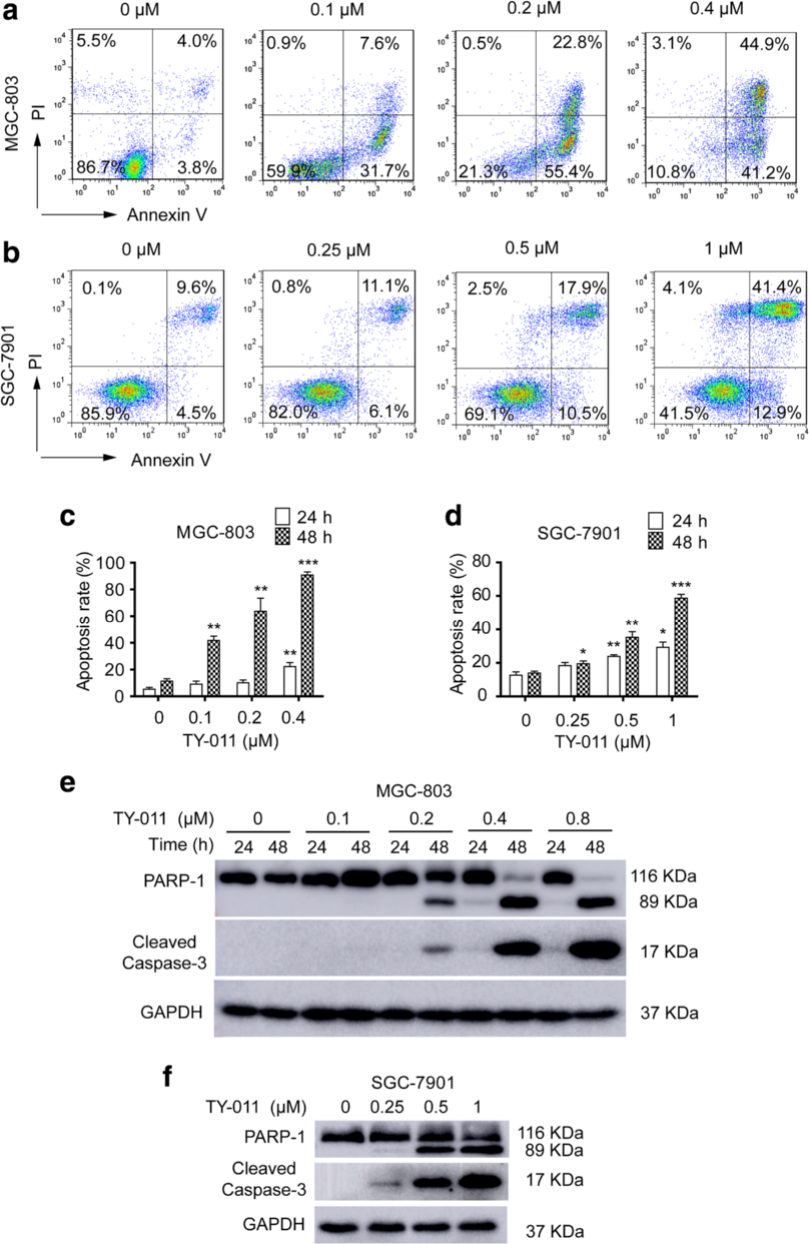
TY-011 induces apoptosis. **a** and **b** MGC-803 and SGC-7901 cells were treated with indicated concentration of TY-011 for 48 h. Apoptosis was assessed by flow cytometry after Annexin V/PI staining. Representative images were shown. **c** and **d** Quantitation of apoptotic cells in **a** and **b**. Data presented are the mean ± SD of three independent experiments. **e** MGC-803 cells were treated with various concentration of TY-011 for the indicated time. **f** SGC-7901 cells were treated with various concentration of TY-011 for 48 h. Cells in (**e** and **f**) were lysed and subjected for western blot to determine the protein levels of cleaved caspase-3 and PARP-1. The results are representative of three independent experiments

We next determined the apoptotic effect of TY-011 in MGC-803 and SGC-7901 cells. Of note, TY-011-induced apoptosis was relatively modest at 24 h, with appropriately 20–30% apoptosis in MGC-803 and SGC-7901 cells treated with the highest concentration (0.4 μM in MGC-803 cells and 1 μM in SGC-7901 cells, respectively). But an obvious increase of apoptosis was observed in both cells at 48 h, which was in a concentration-dependent manner. In detail, compared with 7.8% in control cells, the apoptosis of MGC-803 cells increased to 39.3, 78.2, and 86.1% after treated with 0.1, 0.2 and 0.4 μM TY-011, respectively. Similarly, the apoptotic SGC-7901 cells increased from 14.1 to 17.2, 28.4 and 54.3% after treated with 0.25, 0.5 and 1 μM TY-011, respectively (Fig. 5a–d). Moreover, the cleavage of procaspase-3 and PARP-1, the apoptotic markers, were observed at 48 h after TY-011 treatment both in MGC-803 and SGC-7901 cells (Fig. 5e and f).

### TY-011 suppresses the growth of human gastric xenografts models

**Fig. 6:**
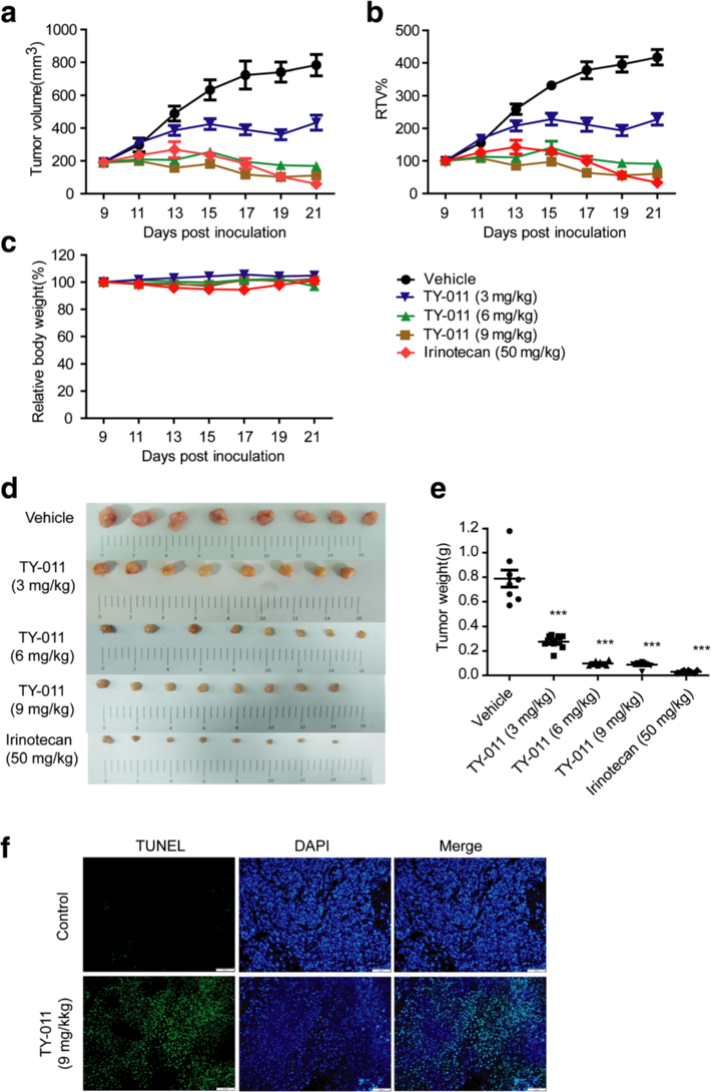
TY-011 suppresses tumor growth in vivo. MGC-803 xenograft bearing nude mice were treated with TY-011 as indicated schedule, respectively. **a** and **b** The tumor volume and RTV were recorded and plotted against days post inoculation. Data presented are the mean ± SEM (*n* = 8). **c** Relative body weight of mice. **d** The pictures of tumors (**e**) Tumor weights. Data presented are the mean ± SEM (*n* = 8). **f** The tumors were harvested and subjected for TUNEL staining to evaluate the apoptosis. Representative images are shown. Scale bar, 50 μm

To further determine the antitumor effect of TY-011 in vivo, we established a subcutaneous gastric carcinoma model using MGC-803 cells. TY-011 was orally administered at 3, 6 and 9 mg/kg once a day for 13 days for MGC-803 tumor-bearing mice. For control tumor-bearing mice, the same amount of vehicle was administered on the same schedule. As shown in Fig. 6a–e and Table 1, tumor growth was significantly inhibited by TY-011 in a dose-dependent manner. The final TGI% of the mice treated with 3, 6 and 9 mg/kg TY-011 were 59.41, 103.77 and 113.09%, respectively. Of note, TY-011 treatment led to tumor regression, 6/8 (75%) for 6 mg/kg group, and 8/8 (100%) for 9 mg/kg group. The average tumor weights of TY-011-treated mice was 0.28, 0.10 and 0.09 g in 3, 6 and 9 mg/kg group, respectively, which were significantly lower than vehicle-treated mice (0.79 g) (Fig. 6e). Meaningfully, TY-011 did not cause drug-related deaths or any loss in body weight of mice (Fig. 6c). In addition, no obvious damages to vital organs (heart, liver, spleen, lung and kidney) or any clinical signs were observed in mice treated with TY-011 (data not shown). These data suggested that TY-011 causes low host toxicity at therapeutic doses.

**Table 1:** In vivo efficacy of TY-011 against human MGC-803 xenograft

Compound	Body weight (g)		Tumor volume (mm^3^)		T/C (%)	TGI (%)	Tumor weight (g)	Inhibition rate (%)
	Start	End	Start	End				
Control	22.1 ± 0.2	22.6 ± 0.2	190.4 ± 6.2	783.7 ± 22.7	/	/	0.79 ± 0.07	/
TY-011 (3 mg/kg)	20.8 ± 0.1	21.8 ± 0.1	192.8 ± 7.1	433.6 ± 16.2***	54.55	59.41	0.28 ± 0.02***	64.9
TY-011 (6 mg/kg)	20.9 ± 0.1	20.3 ± 0.2	191.2 ± 6.2	168.8 ± 7.1***	21.82	103.77	0.10 ± 0.01***	87.7
TY-011 (9 mg/kg)	21.4 ± 0.1	21.7 ± 0.1	189.0 ± 4.9	111.4 ± 3.6***	14.53	113.09	0.09 ± 0.01***	89.0
Irinotecan (50 mg/kg)	21.2 ± 0.2	21.4 ± 0.2	191.7 ± 6.7	59.3 ± 6.5***	8.04	122.31	0.03 ± 0.01***	96.2

The body weight, tumor volume, T/C values, TGI, tumor weight, and inhibition rate were used to evaluate the tumor response to TY-011 treatment

Data presented are the mean ± SEM. ***p < 0.001 *vs*. control

Tumor tissues were then assayed for apoptosis using a TUNEL kit which labels apoptotic nuclei with a fluorescent maker. As shown in Fig. 6f, tumor tissues from animal receiving TY-011 (9 mg/kg) treatment showed an obvious increase in apoptosis. These findings coincided with the apoptotic effect of TY-011 in vitro, highlighting the involvement of apoptosis in the tumor growth inhibitory effects exerted by TY-011 in vivo.

### TY-011 antagonizes the angiogenesis of human endothelial cells

**Fig. 7:**
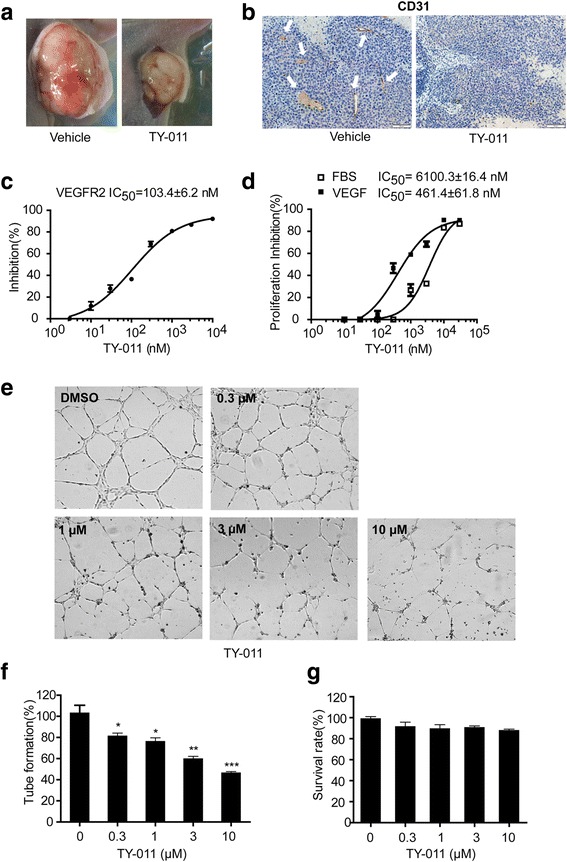
TY-011 antagonizes the angiogenesis. **a** Representative images of tumor tissues. **b** The tumors tissue were sectioned and subjected for immunohistochemistry of CD31 (angiogenesis marker) staining. White arrow means blood vessel. Representative images were shown. Scale bar, 50 μm. **c** The HTRF® KinEASE™-TK kit was used to measure inhibition of VEGFR2 by TY-011. The inhibition rates were determined in reference to the control. Data presented are the mean ± SD of three independent experiments. **d** Growth inhibition effects of TY-011 on HUVECs in the presence or absence of VEGF were determined by CCK-8 assay. Data presented are the mean ± SD of three independent experiments. **e** HUVECs grown on Matrigel^TM^ were treated with various concentration of TY-011 or equivalent DMSO for 6 h. Representative images from three independent assays are shown. **f** Quantitation of tube formation in (**e**). Data presented are the mean ± SD of three independent experiments. **g** HUVECs cells were treated with various concentration of TY-011 for 6 h, and growth inhibition was determined by CCK-8 assay

Interestingly, we observed a significant decreased blood vessel density in tumors from TY-011 treated mice (Fig. 7a), indicating TY-011 might suppress the blood vessel formation. So the tumor tissues were used for immunohistochemistry to visualize the human angiogenesis maker CD31. As shown in Fig. 7b, the CD31 staining in TY-011 treated group was much less than control group, suggesting the vascularization in tumors was inhibited. Then a series of experiments was performed to demonstrate the effects of TY-011 on angiogenesis. Since the activation of VEGFR2 is overwhelmingly regarded as the most critical driver of tumor angiogenesis [28], we first determined the activity of TY-011 against VEGFR2 in vitro. The result showed that TY-011 effectively inhibited the activation of VEGFR2 in a concentration-dependent manner, with IC_50_ of 103.4 ± 6.2 nM (Fig. 7c). To address the specificity of TY-011 antagonizing the endothelial cell growth stimulated by VEGF, HUVECs were exposed to two different stimuli, VEGF and FBS, under the treatment of TY-011 for 72 h. Under these two different culture conditions, HUVECs showed different response to TY-011. The IC_50_ value in cells treated with VEGF were 13-fold lower than in cells treated with FBS; suggesting TY-011 antagonized the HUVECs growth driven by VEGF more potent than by FBS (Fig. 7d). HUVEC tube formation assay were performed to evaluate the effect of TY-011 on tube formation. In DMSO treated group, intact tube meshes was formed. However, these tube meshes were disturbed after exposure to TY-011 for 6 h (Fig. 7e and f). TY-011 didn’t affect HUVEC proliferation under this treatment condition (Fig. 7g), indicating that the effect of TY-011 to antagonize the angiogenesis was not resulted from its toxic effect on HUVEC cells. Together, these results demonstrated that TY-011 had anti-angiogenesis potential.

## Discussion

In the present study, we reported a well-tolerated and orally active small molecule TY-011 for gastric cancer treatment via targeting mitosis pathways and angiogenesis. TY-011 effectively suppressed the activities of Aurora A, Aurora B and VEGFR2 kinases in vitro, inhibited cancer cell proliferation by disturbing mitotic progression and inducing apoptosis, and more importantly, abrogated tumor growth of gastric xenograft model in vivo without any obvious toxicity.

The kinase assay clearly showed that TY-011 concentration-dependently inhibited Aurora A and Aurora B kinases in vitro, with IC_50_ of about hundreds nanomole. Consistently, TY-011 also effectively inhibited the intracellular activities of Aurora A and Aurora B kinases, as evidenced by the decreased phosphorylation of Aurora A (T288) and Histone H3 (S10) in TY-011-treated MGC-803 cells. The molecular docking analysis demonstrated that TY-011 occupied the ATP-binding site of both Aurora A and B kinases. These results indicated that TY-011 is a potent and novel pan Aurora A/B kinase inhibitor.

In line with previously reported pan kinase inhibitors of Aurora A and Aurora B, TY-011 induced an obvious accumulation of cells in G2/M phase and with >4 N DNA content, in a concentration-dependent manner. However, the increase of cells with >4 N DNA content was relatively modest, which might be related with the expression of wild-type p53 in MGC-803 and SGC-7901 cells. It has been reported that whether cells with mitotic defects to be arrested with 4 N DNA content in pseudo-G1 or endoreduplicate with the accumulation of >4 N DNA content depends on the integrity of the p53-dependent postmitotic checkpoint [29]. With the normal function of p53 in both MGC-803 and SGC-7901 cells, the postmitotic checkpoint ensured that most cells with mitotic errors were arrested in tetraploid status.

Importantly, TY-011-treated cells with prolonged mitotic arrest or continued proliferation with cytokinesis failure subsequently underwent apoptosis. Interestingly, TY-011 mainly accumulated cells in G2/M phase and with >4 N DNA content within the first 24 h of treatment, and then induced apoptosis at 48 h. Previous studies have demonstrated that cell death after Aurora A inhibition–mediated mitotic arrest requires activation of proapoptotic pathways [30]. In consistence, here we did not observe the cleavage of procaspase 3 and PARP at 24 h, indicating the activation of proapoptotic pathways happened in the following 24 h, which triggered cell undergo apoptosis. Meaningfully, TY-011-induced apoptosis was also observed in tumor tissues, as evidenced by an increase of TUNEL staining, which is a marker of apoptosis. Therefore, by inducing G2/M phase arrest, polyploidy and apoptosis, TY-011 exhibited its antitumor activity in vitro and in vivo.

Interestingly, we have noticed that tumors in TY-011-treated mice had lower blood vessel density than control group, which was further confirmed by the immunohistochemistry analysis of CD31 maker. Given the important role of VEGFR2 in tumor angiogenesis, we first determined the activity of TY-011 against VEGFR2 kinase. We found that TY-011 was able to inhibit the VEGFR2 kinase with IC_50_ of about hundreds nanomole, effectively inhibit VEGF-stimulated HUVEC cells proliferation and suppress the tube formation of HUVEC cells. These data indicated that TY-011 was able to antagonize tumor angiogenesis. Therefore, TY-011 exhibited efficient antitumor effect by targeting mitotic kinases (Aurora A and B) and angiogenic receptor tyrosine kinase (VEGFR2). As mitosis of cancer cells and angiogenesis by endothelial cells are two key steps of tumorigenesis, we believe that the therapeutic strategy of targeting these two types of cells should be of important value for cancer therapy. Actually, the synergistic antitumor effects of combined inhibition of Auroras and VEGFRs have been receiving much attention. Dual inhibitors of these two types of kinases, such as ENMD-2076, ABT-348, and JNJ-28840172, have been reported recently to exhibit potential antitumor activities in different cancer types [31–33]. Meaningfully, ENM-2076 is currently in Phase I/II clinical trials for several types of cancer [34], which greatly encouraged the development of this type of dual inhibitors. Here we have demonstrated TY-011 exhibited a significant antitumor effect in gastric cancer xenograft model at a very low dose, and showed no observable side effects, therefore TY-011 deserves further investigation and development.

Although the synergistic antitumor effects of targeting Auroras and VEGFRs kinases have been noticed, the mechanism is unclear. Previous studies have demonstrated controlling vascularization of neoplasms by VEGFR inhibition can induce tumor shrinkage, thus causing hypoxic conditions in the tumor environment. Hypoxia stimulates Aurora activity and consequently increases the malignant potential of hepatoma cells [35]. In addition, Aurora A inhibition has been observed to decrease the VEGF transcription and secretion in neuroblastoma cells and inhibit the HUVECs tubule formation [36]. So there might be a feedback loop of Aurora-VEGF-VEGFRs, we presumed that the mechanisms of Auroras and VEGFRs kinases dual inhibitors may be related with cutting off this feedback loop. Besides these three kinases, we could not exclude the possibility that TY-011 might have activities against other kinases or might directly target proteins involved in tumor development. Given the impressive antitumor activity of TY-011 in vivo, better understanding of the mechanisms would definitely beneficial for its development and future application in clinic, and therefore these studies will be performed in our further research work.

## Conclusions

Our study demonstrates that TY-011 is a novel, orally active kinase inhibitor with a mechanism of action that includes inhibition of kinases involved in both mitosis (Aurora A and B) and angiogenesis (VEGFR2). TY-011 exhibits promising antitumor activity both in vitro and in vivo against gastric cancer, and is worthy for further development as a therapeutic approach in gastric cancer.

## Additional file

Additional file 1: Table S1. The inhibitory activities of novel TY derivatives against Aurora A and B kinases and proliferation of MGC-803 cells. **Figure S1.** Time evolution of root-mean-square deviations (RMSD) of backbone atoms during MD simulations. (PDF 224 kb)

## Abbreviations

BCA: Bicinchoninic acid
GC: Gastric cancer
HUVEC: Human umbilical vein endothelial cell
IR: Inhibition rate
MD: Molecular dynamic
MPM2: Mitotic protein monoclonal #2
PME: Particle mesh Ewald
RTV: Relative tumor volume
SAC: Spindle assembly checkpoint
SIE: Solvated interaction energy
TGI: Tumor growth inhibition
TUNEL: Terminal deoxynucleotidyl transferase-deoxyuridine triphosphate nick end labeling
