# Supplementary material for: Antitumor activity of TY-011 against gastric cancer by inhibiting Aurora A, Aurora B and VEGFR2 kinases
Source: J Exp Clin Cancer Res. 2016 Nov 25;35:183. doi: 10.1186/s13046-016-0464-2 (PMC5124248; doi:10.1186/s13046-016-0464-2)
Supplement: Additional file 1: Table S1. — The inhibitory activities of novel TY derivatives against Aurora A and B kinases and proliferation of MGC-803 cells. Figure S1. Time evolution of root-mean-square deviations (RMSD) of backbone atoms during MD simulations. (PDF 224 kb) [file 13046_2016_464_MOESM1_ESM.pdf]

Table S1. The inhibitory activities of novel TY derivatives against Aurora A and B kinase and proliferation of MGC-803 cells

Compounds	IC_{50} (μM)		
	Aurora A	Aurora B	MGC-803
TY-004	>10	1.13	9.64
TY-005	2.36	0.62	2.40
TY-009	>10	1.45	7.98
TY-011	0.10	0.09	0.21
TY-112	7.97	0.78	0.37
TY-123	>10	7.41	>30
TY-145	4.89	0.83	0.21
TY-189	0.33	>10	7.16

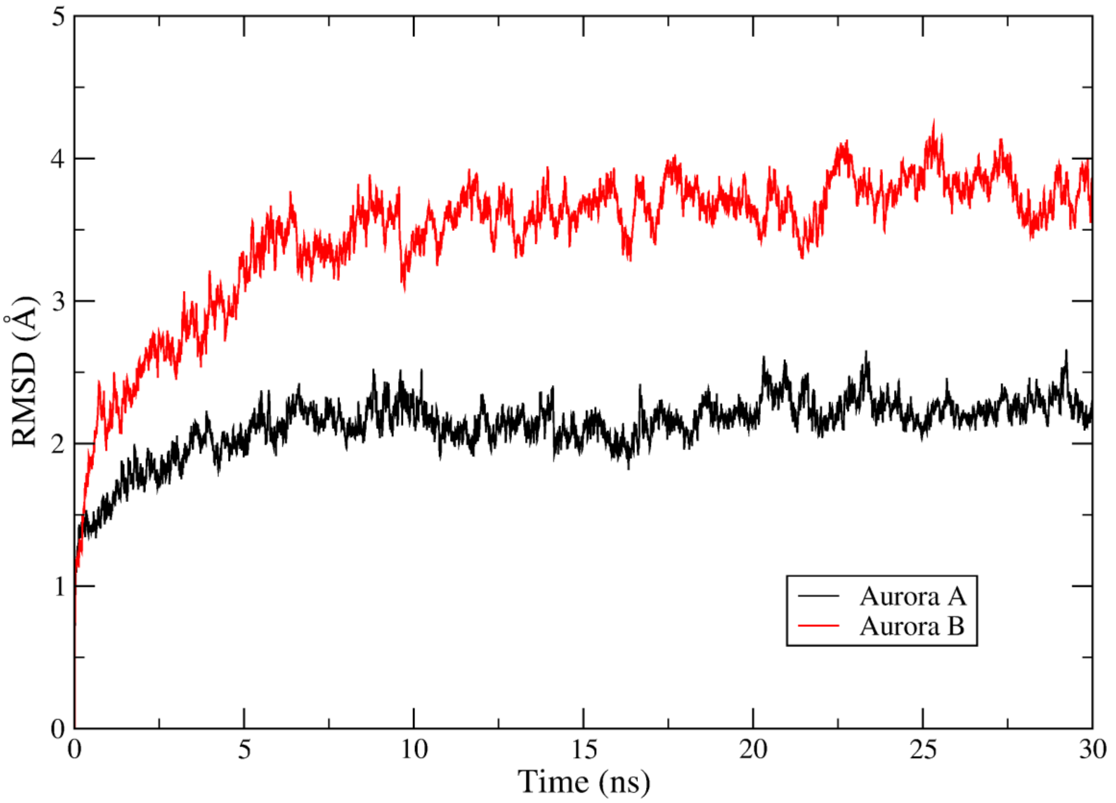

Figure S1. Time evolution of root-mean-square deviations (RMSD) of backbone atoms during MD simulations.
